# The Inflammatory Nature of Post-surgical Delirium Predicts Benefit of Agents With Anti-TNF Effects, Such as Dexmedetomidine

**DOI:** 10.3389/fnins.2018.00257

**Published:** 2018-04-19

**Authors:** Ian A. Clark, Bryce Vissel

**Affiliations:** ^1^Biomedical Sciences and Biochemistry, Research School of Biology, Australian National University, Canberra, ACT, Australia; ^2^Centre for Neuroscience and Regenerative Medicine, Faculty of Science, University of Technology, Sydney, NSW, Australia; ^3^St. Vincent's Centre for Applied Medical Research (AMR), Sydney, NSW, Australia

**Keywords:** delirium, post-operative cognitive deficit, dexmedetomidine, tumor necrosis factor, analgesia, anxiolysis

A characteristic of post-surgery patients, particularly the more elderly, can be a persistent self-propagating cerebral inflammatory syndrome referred to as post-operative cognitive dysfunction (POCD). Changes can be analogous to those seen in Alzheimer's disease (Newman et al., 2007; Steinmetz et al., 2009). Indeed, in some studies the conversion rates to dementia are up to 70% in patients who are 65 years or older (Vanderweyde et al., 2010). An associated transient acute delirium accompanied by increased levels of proinflammatory cytokines, including tumor necrosis factor (TNF), can occur. This sometimes alarming phenomenon can be common in the aged (Inouye et al., 2014), and is often regarded as an extreme manifestation of the sickness behavior caused by cytokines induced during systemic inflammation generated by influences such as trauma or severe infection impinging on a brain vulnerable through already being stressed by these cytokines (Cunningham et al., 2009; Cunningham and Maclullich, 2013; Hennessy et al., 2017).

Recently a report has argued the case that post-surgical delirium can be minimized by prior treatment with dexmedetomidine (Su et al., 2016). Plausible reservations about the form of the trial have been published (Kronzer and Avidan, 2016), and a subsequent trial in which this agent was administered intra-operatively failed to show a response (Deiner et al., 2017). Nevertheless, since a mechanism of action has not yet been suggested, we propose that, should pre-surgical use of dexmedetomidine be confirmed to act against onset of delirium, the capacity of this agent to inhibit excess production of TNF, as demonstrated in various contexts, may well shed light on the field.

Dexmedetomidine (Precedex, Orion Pharma), a synthetic sedative with analgesic and anxiolytic properties, is widely used in surgery. It is a selective α_2_-adrenoceptor agonist that, compared to opiates, causes little respiratory depression. The reported ability of this agent, administered preemptively, to reduce the incidence of post-operative delirium in a large controlled study on elderly patients in intensive care after non-cardiac surgery (Su et al., 2016) may, if confirmed, contain the potential to fill a major need in intensive care units. Questions have since been raised (Avramescu et al., 2017) about whether it confers direct neuroprotective effects or acts indirectly, and its possible mechanism of action, which remains undetermined. However, the rapidly accumulating knowledge on the roles of TNF in brain function draws our attention to a copious literature on interactions between dexmedetomidine and this cytokine. Indeed many have reported on the anti-inflammatory effects of this agent through its effects on this cytokine, as discussed below. In this opinion piece we draw on this literature to explain the proposed inhibitory actions of preemptively administered dexmedetomidine on delirium. The analgesic, anxiolytic and morphine-sparing effects of this agent can also be rationalized in this way. In the first instance, it is useful to note the common pathogenic features of delirium and POCD from a TNF perspective. In 2008 we made the case that the characteristics of the acute illness seen in acute protozoa, bacterial and viral diseases—all of which can all include the extremes of delirium—were formed by the excessive generation of the cytokines released during the phenomenon termed sickness behavior (Clark et al., 2008). Cunningham made essentially the same TNF argument about the pathogenesis of delirium (Cunningham and Maclullich, 2013). We have subsequently extended these arguments in regard to the pathogenesis of POCD (Clark and Vissel, 2015). Moreover, a recent study of post-surgical cognitive impairment has examined the interplay between the human brain and the inflammatory response of the peripheral innate immune system, including the TNF thus generated (Forsberg et al., 2017).

Physical trauma, including that caused by surgery, induces an innate immune response that includes release of pro-inflammatory cytokines such as TNF and interleukins (Arvin et al., 1996). This response follows, in part, from the release of high mobility group box 1 protein (HMGB1) at sites of severe trauma (Cohen et al., 2009). As we have recently discussed in an Alzheimer's disease context (Clark and Vissel, 2015), HMGB1 provides an example of the mechanistic links that can be made between POCD, cytokines, and delirium. A non-histone nuclear protein, HMGB1 is a normal nuclear component of cells. When leaked extracellularly, it can act as a damage-associated molecular pattern (DAMP) molecule that acts as an agonist for toll-like receptor 4 (TLR4), TLR9 and receptor for advanced glycation endproducts (RAGE) on many types of cells, including microglia and astrocytes. This causes the release of pro-inflammatory cytokines, the archetype of which is TNF, which is important in cerebral physiology in low concentrations, and a complex range of pathophysiology when production is excessive (see Clark et al., 2010, for a review).

Since systemic TNF has long been known to cross the blood-brain barrier (Gutierrez et al., 1993), we could expect excess circulating TNF to contribute to cognitive dysfunction (Holmes et al., 2009). It is therefore noteworthy that increased free HMGB1 has been documented to be associated with increased BBB permeability, increased production and presence of TNF in the hippocampus in the cognitive dysfunction of experimental POCD (He et al., 2012). Two groups have recently demonstrated that HMGB1 thus plays an essential part in this model of POCD through ameliorating it with either the HMBG1 antagonist, Box-A (Fonken et al., 2016), or an anti-HMGB1 monoclonal antibody (Terrando et al., 2016). This is consistent with the proposal, based on mouse studies (Terrando et al., 2010), of preventing POCD by preemptively treating at-risk surgical patients with anti-TNF antibody. This body of work on TNF, plus the literature discussed below on interactions between dexmedetomidine and this cytokine, predicts an understanding of how dexmedetomidine, given preemptively, plausibly acts to minimize delirium.

Dexmedetomidine has an extensive history of improving neurological function, for example when given preemptively in animal models tibial fracture (Zhu et al., 2016), sepsis (Qiao et al., 2009), and immediately after the establishment of a brain trauma model in rats (Jiang et al., 2017). In all of these studies, as well as in post-operative treatment of glioma resection patients (Luo et al., 2016), the effect was associated with a reduction in the increased circulating levels of TNF. This agent also has been reported to significantly attenuate microglial activation and TNF production by more than twofold in a mouse model of delayed paraplegia (Bell et al., 2014). An extensive meta-study on its perioperative use (Li et al., 2015) was also associated with a reduction in TNF levels. It is well-documented that TNF is implicated in brain homeostasis, with low levels being essential for normal physiological functioning of cells and synapses. For example, TNF is released during physiological neuronal activity, and plays a crucial role in regulating the strength of normal synaptic transmission (Marin and Kipnis, 2013). It is also involved in normal neurotransmission via modulating excitatory inputs (Pickering et al., 2005), trafficking of AMPA receptors (Ferguson et al., 2008), homeostatic synaptic scaling (Stellwagen and Malenka, 2006; Becker et al., 2013), long-term potentiation (Cumiskey et al., 2007), and control of formation and clearance of synaptic levels of glutamate, a potent toxin when in excess (Clark and Vissel, 2016). Moreover, TNF balance maintains normal background levels of neurogenesis (Bernardino et al., 2008; Russo et al., 2011; Chen and Palmer, 2013). TNF also regulates neuronal type-1 inositol trisphosphate receptors (IP3R), which are central to neuronal Ca^++^ homeostasis, and thus the ionic signaling cascades on which normal function of these cells depends (Park et al., 2008). Clearly, all these functions are vulnerable to TNF being outside its physiological range, with overshoots plausibly being corrected by preemptive use of anti-TNF agents, including dexmedetomidine. Thus neurological function can be expected to diminish when cerebral concentrations of TNF are excessive, with clinical characteristics determined by the local areas where most is present. Importantly, the above reminds us that TNF is biologically much more subtle that merely being a marker for an inflammatory reaction, as often portrayed.

Various pathways of TNF inhibition by dexmedetomidine have been explored. Its action as a α_2_-adrenoceptor agonist appears implicated, in that yohimbine, an α_2_-adrenoceptor antagonist, enhanced TNF levels when the two were compared in a lipopolysaccharide-induced liver damage model (Chen et al., 2015). Dexmedetomidine has also been shown to inactivate the TLR-4/NF-κB pathway through which TNF is commonly induced (Kim et al., 2017). Not surprisingly, therefore, dexmedetomidine reduces TNF generation in carrageenan-induced inflammation (Sukegawa et al., 2014) and also in a myocardial ischemia-reperfusion model (Yang et al., 2017). Evidence also exists that dexmedetomidine potentiates the inhibitory control on TNF release from the vagal anti-inflammatory pathway through the cholinergic pathway (Xiang et al., 2014). In addition, dexmedetomidine inhibits TNF induction by unmethylated CpG DNA, a model for other unmethylated DNA such as that of bacterial or mitochondrial origin (Chen and Qian, 2016). These are strong TNF inducers in bacterial infections and trauma respectively, well-recognized potential inducers of delirium.

Using the same mouse tibial fracture model as did others with dexmedetomidine six years later (Zhu et al., 2016), Terrando and co-workers (Terrando et al., 2010) demonstrated TNF to be the key to post-operative cognitive decline. TNF generation peaked at 30 min post-surgery, and preoperative administration of a specific anti-TNF biological agent greatly ameliorated a standard measure of murine cerebral functional loss (Terrando et al., 2010). By that year this class of therapeutic was already well-established in approved clinical use to treat rheumatoid arthritis, Crohn's disease and ankylosing spondylitis. It has since acquired extensive off-label experience in human cognitive decline states (Tobinick et al., 2012), as well as being successfully employed in an experimental model of stroke (Wu et al., 2016).

Given the pleiotropic nature of TNF, reducing its excess production with dexmedetomidine may also cast light on the mechanisms of other useful outcomes of therapy with this agent that are presently little understood. For instance dexmedetomidine is an acknowledged analgesic, particularly in surgical settings (Vaughns et al., 2017) and in pediatric palliative care (Burns et al., 2017). Excess TNF generates pain (Utreras et al., 2009; Calvo et al., 2012), and reducing TNF in patients (Tobinick and Davoodifar, 2004; Tobinick et al., 2012) or experimentally (Gerard et al., 2015) is reported to reduce pain. Thus the known analgesic properties of dexmedetomidine may reflect its anti-TNF capacity outlined above. We also note that the reported usefulness of dexmedetomidine in cerebral palsy (Liu et al., 2015), a condition characterized by unexplained pain (Fehlings, 2017), may reflect the earlier successful use of etanercept, one of the anti-TNF biological agent in clinical use, in an experimental model of this condition (Aden et al., 2010). Likewise, administering TNF intracerebrovascularly causes overt anxiety in normal mice, whereas etanercept given by the same route is anxiolytic in a mouse model of multiple sclerosis (Haji et al., 2012). Similarly, anxiety states in patients exhibit high proinflammatory cytokine activity (Hou et al., 2017), and dexmedetomidine has anxiolytic properties in rats (Ji et al., 2014). Likewise, both dexmedetomidine (Gursoy et al., 2011) and anti-TNF agents (Shen et al., 2011; Sun et al., 2012) attenuate the expression of the tolerance to morphine that develops with its continued use in chronic pain.

The background information required to rationalize the contrasting outcomes reported in the two trials (Su et al., 2016; Deiner et al., 2017) that are the basis of this opinion piece is as follows. In summary, the trauma associated with surgery rapidly releases HMBG1 and mitochondrial DNA from damaged cells. These are strong DAMPs that activate TLRs to generate inflammatory cytokines in harmful excess. TNF, the first cytokine in the inflammatory cascade, is released, and cleared, most rapidly. Thus it has already initiated many pathways of pathophysiology, including in the brain (since these cytokines cross the blood-brain barrier, Banks et al., 1995). The literature on the inhalation anesthetics also inducing TNF also warrants briefly acknowledging here (Wu et al., 2012). The observation of anti-TNF antibody being administered to baboons 2 h before an LD100 of *Escherichia coli* protecting them completely from harm (Tracey et al., 1987) is in sharp contrast to the uselessness of neutralizing TNF once clinical sepsis is underway (Fisher et al., 1996).

Thus it seems logical that, in the context of post-surgical delirium (Su et al., 2016; Deiner et al., 2017), dexmedetomidine is likely to be acting by inhibiting TNF production, its efficacy in these two studies determined by the timing of its administration in relation the onset of the surgical event. When given beforehand, whether the event is delirium (Su et al., 2016) or sepsis (Tracey et al., 1987), TNF's effects can be nipped in the bud. In contrast, once the acutely harmful clinical event, be it delirium (Deiner et al., 2017) or sepsis (Fisher et al., 1996) is in train, the TNF already released has initiated harmful events, so it is too late to expect to reverse them by neutralizing this cytokine.

A useful step in understanding its mechanism further would be to experimentally compare preemptive use of dexmedetomidine and one of the specific anti-TNF biologicals reported to minimize POCD delirium, pain and anxiety, and to induce morphine tolerance. Because of their molecular size, these biologicals would require administering intracerebroventricularly or perispinally (Tobinick, 2007), whereas the routine use intravenous of the small molecule dexmedetomidine as a sedative infers its brain entry after systemic administration. This comparison could lead to preemptive anti-TNF biologicals being a very much more rational and effective therapeutic than dexmedetomidine in this context.

## Author contributions

IC proposed the scope of the review. Both authors were involved in planning and editing the manuscript, blending their complementary expertises. Both authors read, altered and approved the final manuscript.

### Conflict of interest statement

The authors declare that the research was conducted in the absence of any commercial or financial relationships that could be construed as a potential conflict of interest.
